# Loop-Mediated Isothermal Amplification in a Core-Shell Bead Assay for the Detection of Tyrosine Kinase AXL Overexpression

**DOI:** 10.3390/mi12080905

**Published:** 2021-07-30

**Authors:** Kamalalayam Rajan Sreejith, Muhammad Umer, Pradip Singha, Nhat-Khuong Nguyen, Surasak Kasetsirikul, Chin Hong Ooi, Muhammad J. A. Shiddiky, Nam-Trung Nguyen

**Affiliations:** 1 Queensland Micro- and Nanotechnology Centre, Griffith University, 170 Kessels Road, Nathan, QLD 4111, Australia; s.kamalalayamrajan@griffith.edu.au (K.R.S.); m.umer@griffith.edu.au (M.U.); pradip.singha@griffithuni.edu.au (P.S.); nhatkhuong.nguyen@griffithuni.edu.au (N.-K.N.); surasak.kasetsirikul@griffithuni.edu.au (S.K.); c.ooi@griffith.edu.au (C.H.O.); 2 School of Environment and Science, Nathan Campus, Griffith University, 170 Kessels Road, Nathan, QLD 4111, Australia

**Keywords:** loop-mediated isothermal amplification, AXL overexpression, core-shell bead assay

## Abstract

The upregulated expression of tyrosine kinase AXL has been reported in several hematologic and solid human tumors, including gastric, breast, colorectal, prostate and ovarian cancers. Thus, AXL can potentially serve as a diagnostic and prognostic biomarker for various cancers. This paper reports the first ever loop-mediated isothermal amplification (LAMP) in a core-shell bead assay for the detection of AXL gene overexpression. We demonstrated simple instrumentation toward a point-of-care device to perform LAMP. This paper also reports the first ever use of core-shell beads as a microreactor to perform LAMP as an attempt to promote environmentally-friendly laboratory practices.

## 1. Introduction

The receptor tyrosine kinase AXL—initially identified to be overexpressed in human myeloid leukemia cells—has, over the years, emerged as a promising diagnostic and therapeutic target for a range of cancers [1]. The AXL receptor is activated by the binding of ligand growth arrest-specific gene-6 (GAS6). Several major cellular signaling pathways, such as PI3K-AKT-mTOR, NF-kB and JAK/STAT, are activated downstream of the GAS6-AXL binding event, indicating the pivoting role that AXL plays in normal cellular functioning [2]. AXL signaling is involved in cell proliferation and survival, cell adhesion and migration, and the regulation of inflammatory cytokines. AXL is overexpressed in several cancers, including gastric, breast, colorectal, prostate and ovarian cancers. The overexpression of AXL has been found to be involved in angiogenesis, resistance to chemotherapy, and attenuated antitumor immune response. AXL also plays an important role in epithelial to mesenchymal transformation (EMT), and cell invasion and migration, highlighting its role in cancer metastasis. As a result, AXL has been extensively explored not only as a therapeutic target but also as a diagnostic and prognostic marker [1,2].

Among molecular diagnostic tools, isothermal amplification methods have attracted widespread interest in recent years as possible alternatives to the gold standard polymerase chain reaction (PCR)-based analyses [3,4] As these isothermal amplification reactions are carried out at a constant temperature, the reliance on sophisticated thermal cyclers can be eliminated. Therefore, these isothermal amplification methods can prove to be an attractive approach for the development of point-of-care molecular analysis platforms. Among the various isothermal amplification methods, loop-mediated isothermal amplification (LAMP) is the most widely used technique [4]. LAMP offers several advantages, such as high specificity due to the use of four to six primers, tolerance to inhibitors, and the ability to directly detect RNA without the requirement of a reverse transcription step. Taken together, LAMP appears to be a suitable method of choice for point-of-care applications [5]. However, despite the simplicity of the procedure, the detection of the amplified product is still a significant challenge. Although naked-eye colorimetric or turbidimetric methods for the detection of LAMP products have been widely used, these methods provide qualitative information at best. In situations where the accurate quantification of the target may be required, such as the monitoring of the expression level of cancer-related genes such as AXL, a more robust detection method may be needed. The fluorescent detection of LAMP amplicons may be more appropriate in this case; however, it requires sophisticated instrumentation.

All molecular diagnostic tools, including LAMP, are currently carried out in conventional plastic vials or conventional microfluidic chips. Approximately 5.5 million tons of plastic waste was reported to be generated only from biolabs around the world in 2014 [6]. Conventional molecular diagnostic methods, such as PCR and LAMP, add a significant contribution to this. The recent development of microfluidics and microtechnologies allows for the integration of flow control [7] and temperature control [8,9] on a chip, making the compact implementation of PCR and LAMP possible. However, conventional microfluidic chips are not a solution for the reduction of the non-reusable plastic waste they generate. Droplet-based microfluidics has a great potential for the reduction of sample size and using temperature control not only for the reaction but also for droplet actuation [10]. This paper reports the use of core-shell beads as a droplet-based microreactor to carry out LAMP reactions. Core-shell beads belong to the emerging field of micro elastofluidics [11], consisting of a core liquid embedded in a transparent, hard, spherical shell. Core-shell beads are one promising replacement for conventional plastic vials and microfluidic chips. Core-shell beads could reduce the amount of plastic waste by 86% [12]. A Core-shell bead was also demonstrated to serve as a microreactor for PCR [12,13]. However, to the best of our knowledge, no study has been reported to utilize core-shell beads as a microreactor for LAMP. Furthermore, no study has been reported on the LAMP of AXL genes as a potential candidate for the diagnosis of certain cancers. The present paper reports the use of LAMP in core-shell beads to detect AXL overexpression as a potential diagnostic tool for cancer detection.

## 2. Materials and Methods

### 2.1. Cell Culture and Isolation of RNA

The immortalized human mesothelial cell line MeT-5A was purchased from the American Type Culture Collection (ATCC), VA, Manassas USA. The cells were cultured in T-25 cell culture flasks using RPMI 1640 medium supplemented with 10% heat-inactivated fetal bovine serum (FBS), 100 U/mL penicillin and 100 mg/mL streptomycin. The cultures were maintained in a humidified incubator at 37 °C with 5% CO_2_. The cells were harvested by trypsinization at 70–80% confluence, and pelleted by centrifuging at 2000× *g* for 5 min. The RNA was isolated from the pelleted cells using a Monarch total RNA miniprep kit (Cat # T2010S, New England Biolabs, Notting Hill, Australia) following the manufacturer’s protocol. The purified RNA was finally eluted in nuclease-free water. The quality and quantity of the RNA were analyzed using a NanoDrop spectrophotometer.

### 2.2. Preparation of the LAMP Reaction Mixture

A 214 bp-long synthetic target corresponding to the positions 1688–1901 of AXL transcript variant 2 in the mRNA reference sequence (NM_001699.6) was used as a template to optimize the reaction conditions. Table 1 lists the template and primer sequences. The target sequence was isothermally amplified using a WarmStart^®^ LAMP Kit (DNA and RNA), (Cat # E1700S, New England Biolabs, Notting Hill, Australia) in a 10 µL reactor, as per the manufacturer’s instructions. The reaction mixture consisted of 5 µL WarmStart LAMP 2× Master Mix, 0.2 µL 50× fluorescent dye, and 1 µL 10× primer mix (final concentrations: FIP/BIP 1.6 µM, F3/B3 0.2 µM). A volume of 1 µL the synthetic target of the known copy number was used, and the reaction was made up to 10 µL using nuclease-free water (Cat # 10977015, Thermo Fisher Scientific, Scoresby, Australia). For the cell line LAMP, 100 ng of the total RNA isolated from Met-5A cells was used as a template. The real-time monitoring of the change in the fluorescence levels during the LAMP reaction was carried out in parallel on a CFX96 Touch Real-Time PCR Detection System (Bio-Rad, Gladesville, Australia), in order to compare the functionality and efficiency of LAMP in core-shell beads. The samples were incubated at 65 °C for 45 min, and the fluorescence signal was recorded in the FAM channel after every minute. The reaction was stopped by denaturing the Bst 2.0 and RTx enzymes at 85 °C for 5 min. The melt curve analysis of the amplified products was carried out by heating for 5 s each between 65 °C and 95 °C at 0.5 °C increments. The data collection was enabled at each increment of the temperature.

### 2.3. Preparation of the Core-Shell Beads

The core-shell beads required for this experiment were made using liquid marble technology. Liquid marbles are liquid droplets coated with fine particles of hydrophobic or oleophobic powder [14]. Liquid marbles find applications in cell biology, sensing, disease diagnosis and pathogen detection [15,16,17]. Liquid marbles also serve as a microreactor platform to carry out biochemical reactions [18,19,20]. However, the evaporation of liquid marbles at elevated temperatures limits their use in reactions carried out at elevated temperatures [21,22,23,24,25]. The liquid marble technology can be used to synthesize core-shell beads with a hard shell. A hard shell would prevent evaporation. In this experiment, we used a photopolymer as the shell liquid and the LAMP reaction mixture as the core liquid.

Amounts of 0.05 g camphorquinone and 0.06 g ethyl-4-(dimethylamino) benzoate in 10 g Trimethylolpropane trimethacrylate (TRIM) were mixed well in a glass beaker using a magnetic stirrer at 600 rpm for 2 min. The prepared photopolymer mixture was stored in an opaque container for future use. Trimethylolpropane trimethacrylate is a crosslinking monomer. To the best of our knowledge, no cytotoxicity has been reported for TRIM, and this compound has been used frequently in dentistry, and for making bone cement [26,27].

A super amphiphobic silicon monolith called “marshmallow-like gel” (MG) [28] was used as the oleophobic material for the synthesis of core-shell beads. The MG was received from one of our collaborators at Kagoshima University, Japan. The delicate thin sheets of MG were powdered using a mortar and collected in an open plastic plate (35 mm × 35 mm × 10 mm), forming a super-amphiphobic powder bed.

**Figure 1:**
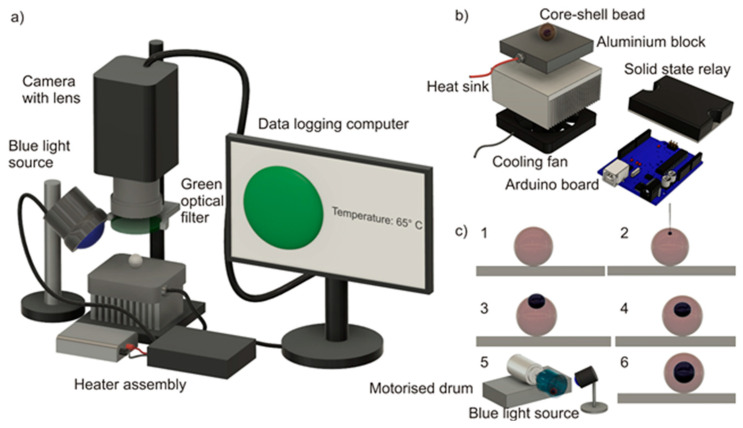
Core-shell bead-based LAMP: (**a**) experimental setup; (**b**) exploded view of the heater platform. (**c**) Various stages of core-shell bead synthesis.

Various steps involved in the synthesis of the core-shell bead using liquid marble technology are depicted in Figure 1c. In step 1, 20 µL of the previously prepared photopolymer liquid was deposited onto the amphiphobic powder bed. In step 2, 2 µL LAMP mixture was injected into the photopolymer liquid droplet using a micropipette. In step 3, the LAMP mixture was observed to be partially immersed in the photopolymer, exposing almost one-third of its volume to the environment. The photopolymerization of the outer liquid at this stage will not be efficient, as the sample liquid may evaporate during the heating of the bead. In step 4, the composite liquid phase obtained from step 3 was gently rolled over the powder bed to coat the droplet with the powder, and to generate a composite liquid marble. Composite liquid marble is a liquid phase (core liquid) covered with another liquid phase (shell liquid), which is again covered with a solid phase (amphiphobic powder). We observed that a one-third portion of the core liquid which was exposed to the ambient was just subsided beneath the exterior photopolymer liquid after coating it with the powder. This is due to the higher “phobic” nature of the core liquid to the powder than the photopolymer liquid. Photopolymerization at this stage will result in a core-shell bead. However, the thickness of the shell above the core liquid will be relatively thin, and may be prone to breakage due to thermal stress during the heating process. It is better to position the core liquid to the center of the photopolymer to avoid this problem. In step 5, the composite liquid marble obtained after step 4 was transferred to a motorized cylindrical drum. The motorized drum was rotated at 140 rpm for 5 min with a blue light source kept at 5 cm above it, continuously illuminating the rotating composite liquid marble. It has already been reported that the rotary motion of the composite liquid marble would push the core liquid drop to the center of the exterior liquid [29]. In step 6, the process of simultaneous rotation and photopolymerization resulted in core-shell beads of which the core liquid is relatively perfectly at the center of the hardened shell. The core-shell bead obtained would be covered with amphibhobic powder. This can be cleaned by washing them in deionized water, resulting in transparent core-shell beads.

### 2.4. Design and Fabrication of the Isothermal Heater and Fluorescent Monitoring System

A commercial thermal cycler and fluorescent monitoring system for core-shell bead-based LAMP are not available. Hence, we developed a customized thermal cycler and fluorescent monitoring system. An aluminum block (20 mm × 20 mm × 15 mm) embedded with a cartridge heater (5 mm diameter and 15-mm length, Core electronics) was used as the heating platform. The power to the heater was controlled using a PID (proportional-integral-derivative) algorithm implemented in an Arduino UNO microcontroller board. The real-time temperature of the heater block was monitored by an LM 35 temperature sensor glued to the aluminum block and fed back to the Arduino board. The temperature setpoint was 67 °C (a +2K offset is provided to compensate for the temperature difference between the heater platform and the core-shell bead). The entire heater assembly was attached to an aluminum heat sink (85.6 mm × 68.3 mm × 41.5 mm) and a cooling fan assembly (12 V, 3300 rpm, 70 mm × 70 mm × 25 mm). The heat sink and cooling fan assembly were implemented for safety purposes. The cooling fan will be activated and the power supply to the heater block will be disconnected if the temperature of the heater block exceeds 90 °C, thus protecting the device from thermal runaway.

The excitation and emission spectra of the LAMP mixture were 450–490 nm (blue) and 520–560 nm (green), respectively. A customized excitation light source was formed by 30 circularly arranged blue LEDs. The excitation light source was powered by a 12 V, 3 A DC adapter. A vertically mounted CMOS camera (Edmund Optics EO-5012C, Edmund optics, Barrington, NJ, USA) attached to a 0.5× telecentric lens (Edmund Optics-63074, Edmund optics, Barrington, NJ, USA) and a green optical filter (520–560 nm) was used as the fluorescent detection device. The excitation source was automatically switched on at intervals of 10 min, and the images of the fluorescence emissions were captured simultaneously. The intermittent fluorescent excitation (with an interval of 10 min) was chosen to avoid the photobleaching of the sample. Figure 1a depicts the experimental setup. Figure 1b shows the exploded view of the thermal cycler assembly. The custom-built thermal cycler demonstrated a ramping rate of 0.68 K/s during the heating, and a steady-state error of ±0.5 K.

**Figure 2:**
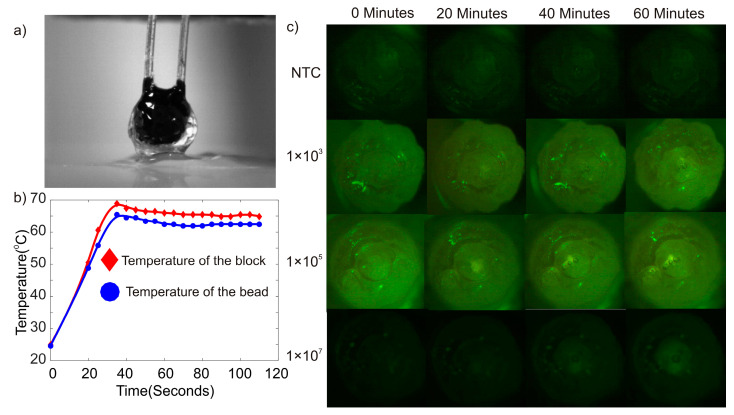
(**a**) Photograph of a bead embedded with a thermistor. (**b**) Temperature reading of the bead and the heater block. (**c**) Photographs of the core-shell beads at various times during the LAMP reaction.

An additional dummy experiment was carried out to check whether the core-shell bead possesses the same temperature as the heater platform. In total, 50 µL photopolymer liquid was dropped onto the oleophobic powder bed, and a thermistor (NTC-10) was inserted into the liquid photopolymer drop. The droplet embedded with the thermistor was subsequently illuminated with blue light for 5 min. The photopolymerization of the liquid droplet provided a hardened bead embedded with the thermistor. This bead was subsequently placed on the heater platform. The platform was heated to 65 °C, and the temperatures of the heater platform and the bead were noted. There was an average temperature difference of 2.12 K between the heater platform and the bead. Figure 2a shows the image of the bead embedded with the thermistor. Figure 2b depicts the thermal characteristics of the heater platform and the bead.

### 2.5. Experimental Procedure

The first phase of the experiment was completed using a synthetic DNA template corresponding to AXL mRNA RefSeq. Three different starting template copy numbers (C. Ns)—1 × 10^3^, 1 × 10^5^, and 1 × 10^7^—were tested. A no-template control (NTC) reaction was also included. The reactions were carried out on a real-time PCR machine.

The second phase of the experiments was completed using a core-shell bead as a microreactor, and in a specially developed thermal cycler. The core-shell beads were prepared using 20 µL photopolymer liquid and 2 µL sample mixture, as described in Section 2.3. Core-shell beads containing three different starting template copy numbers—1 × 10^3^, 1 × 10^5^, and 1 × 10^7^—were prepared. The core-shell beads were placed onto the isothermal heater below the telecentric lens of the fluorescent monitoring system, Figure 1a. The isothermal heater was preheated to 67 °C instead of 65 °C to compensate for the temperature difference between the heater platform and the bead, as noted in the dummy experiment. Samples containing NTC were subjected to LAMP for 60 min in order to observe whether any amplification occurred. Observing a negative amplification from samples containing NTC after 60 min, we decided to carry out the reactions for the core-shell beads for 60 min instead of 45 min. This would provide a window for the reaction to be successful for a sample volume of 2 µL in a core-shell bead compared to a sample volume of 10 µL in a conventional machine. The experiments were repeated three times for each starting template concentration, and for the core-shell beads containing NTC.

We carried out the LAMP reactions using Met-5A total RNA under similar reaction conditions. The total RNA isolated from the cells was diluted to a concentration of 100 ng/µL using nuclease-free water. A volume of 1 µL total RNA was used as a template in the LAMP reaction. The secondary phase of the experiment was completed using a cell line sample.

### 2.6. Image Processing and Data Analysis

Images of the core-shell beads under heating were taken periodically every 10 min for 60 min. The average pixel density of the circular region containing the sample was obtained using Image J software. This average pixel density was considered as the numerical equivalent of the fluorescence emitted from the core-shell bead. This numerical equivalent of the fluorescence was subsequently offset corrected and normalized to minimize the disparity among the results, and to provide a better comparative study. The offset correction and normalization were implemented according to the following equation:

(1) $$I_{st}^{*}=\left(I_{st}-I_{s0}\right)/I_{max}$$

where, $I_{st}$ is the numerical equivalent of the fluorescent intensity of the sample at any instant, $I_{s0}\;$ is the numerical equivalent of the fluorescent intensity of the corresponding sample at the beginning of the LAMP reaction, and $I_{max}$ is the numerical equivalent of the maximum fluorescent intensity obtained among all of the samples tested. The standard error of the readings was calculated as

(2) $$S.E=\sigma /\sqrt{n}$$

(3) $$\sigma =\sqrt{\frac{\sum {\left(I_{st}^{*}-I_{st_{mean}}^{*}\right)}^{2}}{n-1}}$$

where $I_{st\_mean}^{*}$ is the average of the numerical equivalent of the fluorescent intensities of various samples with the same template concentration at an instant.

## 3. Results and Discussion

**Figure 3:**
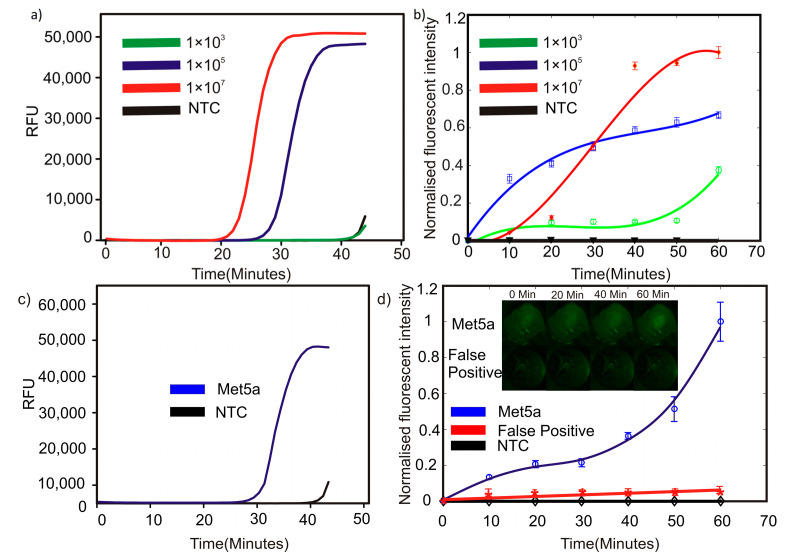
LAMP results: (**a**) synthetic target on a conventional qPCR platform, (**b**) synthetic target in cores-shell beads, (**c**) Met5a RNA on a conventional qPCR platform, and (**d**) Met5a RNA in core-shell beads.

Figure 3a shows the amplification of the LAMP reactions as the fluorescent signals (relative fluorescence unit, RFU) obtained from each sample versus time in a conventional commercial thermal cycler. Out of the three tested concentrations of the synthetic target, the primers used in this study could amplify only the templates with up to a 1 × 10^5^ starting copy number. No amplification was observed at up to 40 min of incubation with a template with 1 × 10^3^ copies as the starting material. Similarly, we observed no amplification for the NTC samples for up to 40 min. Both the 1 × 10^3^ sample and the NTC showed a little amplification around 43 min. However, considering the low starting template concentration, and that NTC showed a similar amplification trend, the detection limit of our assay in a conventional thermal cycler was 1 × 10^5^ the template copy number.

Figure 3b depicts the amplification obtained from the LAMP reaction carried out in core-shell beads. In contrast to the results obtained from the LAMP reaction carried out in a conventional machine, the sample containing 1 × 10^3^ starting copies showed a fluorescence emission after 20 min. Subsequently, the fluorescence grew steadily until 50 min. A sudden surge of fluorescence was observed after 60 min. This behavior could be considered as positive fluorescence, as the samples containing NTC did not show any fluorescence after 60 min. The samples containing an initial copy number of 1 × 10^5^ and 1 × 10^7^ showed a steady increase in fluorescence from the beginning to the end of the reaction. The end fluorescent intensity of the samples followed a logical pattern of 1 × 10^7^ > 1 × 10^5^ > 1 × 10^3^. It should be noted that the fluorescent intensity values of the samples containing initial template copy numbers of 1 × 10^5^ and 1 × 10^7^ already reached more than 80% of their corresponding maximum values after 45 min, provided that only 2 µL of the sample was used compared to LAMP in a conventional plastic vial. However, it is clear that a direct comparison of the results from plastic vial-based conventional LAMP with core-shell bead LAMP is not possible due to the difference in the fluorescence detection methods and algorithms; the trend seen in the outcomes of the core-shell bead-based LAMP can be considered as a promising indication of them for consideration as an alternative for conventional methods. Our assay could detect a concentration as low as 1 × 10^5^ of the starting copies on a conventional thermocycler. However, the assay sensitivity increased to about 500-fold in core-shell beads, considering that the reactions for core-shell beads were prepared initially in 10 µL, out of which only 2 µL were used. Thus, the detection limit achieved on the core-shell beads was about 200 starting template copies. Although LAMP assays capable of detecting DNA molecules down to single copy numbers have been reported, particular sets of primers may be susceptible to false positives at low template concentrations [30].

Although the determination of the efficiency of the LAMP assay is beyond the scope of this paper, further improvements for the sensitivity of our platform are possible through the careful optimization of the LAMP primers, especially the inclusion of loop primers.

We further demonstrated the feasibility of the LAMP assay for the use of total RNA isolated from the Met-5A cell line. Met-5A is a human mesothelial cell line immortalized by the transformation of epithelial virus SV40. Met-5A expresses a high level of AXL RNA [31]. To the best of our knowledge, we described for the first time, here, a LAMP assay for the detection of AXL in complex biological samples. AXL is an oncogene that is involved in several cancer-promoting processes, and is found to be overexpressed in a variety of cancers. AXL has emerged as a promising therapeutic target, and several AXL inhibitors are currently in clinical trials [1]. Therefore, the development of assays for AXL detection may be useful for pan-cancer diagnostics and theragnostics. We demonstrated that our platform can specifically detect AXL in a complex mixture of cellular RNA. The RFU value for isothermal AXL amplification using Met-5A-derived total RNA crossed the threshold at around 32 min on the conventional thermal cycler. However, using core-shell beads, a high fluorescent signal was observed as early as 10 min from the start of the experiment. Amplification was observed for Met5a RNA at around 32 min. Effective heat transfer between the heater platform and the sample inside the core-shell bead, and a small sample volume in core-shell beads, might be the possible factors leading to the improved amplification efficiency in core-shell beads. However, further research must be extended in this direction to gain more insight. The propriety fluorescent dye used in the NEB LAMP kit binds non-specifically to dsDNA. During a nucleic acid amplification reaction such as LAMP, non-specific dsDNA molecules may be generated. These dsDNA molecules correspond to the possible primer-dimers or products generated from the amplification of non-target templates. The amplification from non-target templates is a major concern when using complex biological samples such as total cellular RNA. In order to verify that the fluorescent signal observed in our LAMP in the core-shell bead assay is from the specific amplification of the target, and to study the effect of possible non-specific amplification on the overall fluorescence signals, we carried out a control LAMP reaction with non-specific primers. The so-called “unrelated primers control” (UPC) reaction used Met-5A total RNA as a template, and a primer set which was not targeted against any human gene (Table 2) [32]. As indicated in Figure 3d, the background fluorescence signal from the non-specific dsDNA molecules generated during the LAMP reaction was less than 6% of the maximum fluorescence signal obtained from the positive sample, and it is negligible to be considered as negative.

Although the core-shell bead-based LAMP assay demonstrated a fluorescent output both at high and low template concentrations, the amplification curve of the samples containing 1 × 10^7^ template copies showed a lower fluorescence than those containing 1 × 10^5^ after 30 min. This discrepancy can be attributed to a few factors. First, the synthesis of the core-shell bead was not optimized in terms of the quality of the core-shell bead. Due to this reason, the position of the core liquid could be different inside the shell in each bead. The geometric location of the core sample liquid plays an important role in the fluorescence emitted from the bead. Second, the transparency of the shell material could be another issue. Even though the oleophobic powder is washed away from the bead after its synthesis, some residue of the powder may remain on the core-shell bead surface, affecting the fluorescence reading. Third, the lighting and fluorescence detection system was made from off-the-shelf components. Even a minute change in the illumination system can affect the fluorescence output. However, this discrepancy in the fluorescent intensity values at a time before the total reaction time of the LAMP is almost irrelevant, as LAMP is mostly used for endpoint detection.

## 4. Conclusions

The loop-mediated isothermal amplification (LAMP) of 10-µL samples containing synthetic AXL genes was carried out in conventional plastic vials and a thermal cycler. Successful amplification was recorded for samples containing 1 × 10^5^ and 1 × 10^7^ initial template copies. Samples containing 1 × 10^3^ initial copies did not show any amplification. The Met5a cell line was also tested, yielding a positive amplification in conventional methods after 30 min. The subsequent experiments in core-shell beads using 2-µL sample volume in a specially developed thermal cycler yielded positive results. In contrast to the conventional method, samples containing 1 × 10^3^ initial copy numbers demonstrated amplification in core-shell beads after 20 min, and exhibited a peak fluorescence after 60 min. The samples containing copy number templates 1 × 10^5^ and 1 × 10^7^ also demonstrated amplification, and the endpoint fluorescence followed a pattern of 1 × 10^7^ > 1 × 10^5^ > 1 × 10^3^. A subsequent experiment using a Met5a cell line in a core-shell bead demonstrated amplification after 10 min of heating. The fluorescent intensity was more than 10% of its maximum value after 10 min. It gave a fluorescent intensity of more than 30% of its maximum value after 40 min, and the peak fluoresce was observed after 60 min of heating. Furthermore, we demonstrated the detection of AXL gene overexpression using loop-mediated isothermal amplification. We also demonstrated that the reaction can be carried out in core-shell beads instead of conventional plastic vials or a microfluidic device. The spherical shape of core-shell beads could provide a simple solution for focusing and detecting light [33] that potentially further simplifies the design of the optical system. The simplicity of designing and developing a thermal cycler, as well as of the image detection hardware and the image processing algorithm, provides a larger window for engineering opportunities to develop environmentally friendly point-of-care diagnostics for the detection of cancer using LAMP in core-shell beads.

## Figures and Tables

**Table 1:** LAMP template and primer sequences.

Name	Sequence
AXL_F3	CCTGGGCATCAGTGAAGAG
AXL_B3	CGCTTCACTCAGGAAATCCT
AXL_FIP	TCCAAACTCTCCCTCTCCCAGATTTTGGGATGTGA
	TGGTGGACCG
AXL_BIP	ATGGAAGGCCAGCTCAACCAGTTTTCCTCGTGCAG
	ATGGCAATC
Synthetic target	CCTGGGCATCAGTGAAGAGCTGAAGGAGAAGCTG
	CGGGATGTGATGGTGGACCGGCACAAGGTGGCCC
	TGGGGAAGACTCTGGGAGAGGGAGAGTTTGGAGC
	TGTGATGGAAGGCCAGCTCAACCAGGACGACTCCA
	TCCTCAAGGTGGCTGTGAAGACGATGAAGATTGCC
	ATCTGCACGAGGTCAGAGCTGGAGGATTTCCTGAG
	TGAAGCG

**Table 2:** Primer sequences for the UPC reaction.

Name	Sequence
Cor-RdRp-F3	GCTCGCAAACATACAACGT
Cor-RdRp-B3	GTTACCATCAGTAGATAAAAGTGCA
Cor-RdRp-FIP	CGCCACACATGACCATTTCACTCAATTTTGTTGTAGCTTGTCACACCGT
Cor-RdRp-BIP	AGGTGGAACCTCATCAGGAGATGTTTTAACATTGGCCGTGACAGC
Cor-RdRp-LF	CTTGAGCACACTCATTAGCTAATC
Cor-RdRp-LB	CCACAACTGCTTATGCTAATAGTGT
